# Infectious role of periapical abscesses and its influence on healing outcomes

**DOI:** 10.3205/dgkh000592

**Published:** 2025-11-07

**Authors:** Karthik Shunmugavelu, Vimala Rani Swaminathan

**Affiliations:** 1 Department of Dentistry, PSP Medical College Hospital and Research Institute, Oragadam Panruti Kanchipuram, district Tamil Nadu, India; 2 Department of Physiology, Sree Balaji Medical College and Hospital, Chromepet, Chennai, Tamil Nadu, India

**Keywords:** intracanal medications, calcium hydroxide, triple antibiotic paste, nanoparticles, Ledermix paste, postoperative pain, apical periodontitis, cone beam computed tomography, endodontics

## Abstract

**Background:**

Post-treatment apical periodontitis and postoperative pain remain critical challenges in endodontics, with success often relying on effective intracanal medications to control microbial infection, reduce inflammation, and promote tissue healing. Recent advances in medication formulations and techniques have expanded the options for managing these conditions, necessitating a systematic review of their efficacy. Thus, this review evaluated the effectiveness of various intracanal medications, including calcium hydroxide (CH), triple antibiotic paste with diclofenac potassium (TAPC), nano-based formulations, and Ledermix paste, in reducing microbial load, controlling inflammation, and alleviating postoperative pain in endodontic treatment.

**Method:**

Relevant studies were reviewed to assess the antimicrobial efficacy, pain management outcomes, and healing potential of different intracanal medications. The studies utilized various methodologies, including randomized controlled trials and retrospective analyses, with outcome measures such as bacterial reduction, levels of inflammatory mediators, pain scores, and imaging-based assessments of lesion healing.

**Results:**

CH demonstrated significant microbial reduction (up to 99.5%), decreased inflammatory mediators (IL-1β, TNF-α), and enhanced tissue healing, making it a reliable choice for managing apical periodontitis. TAPC showed superior pain relief compared to CH due to its dual antibacterial and anti-inflammatory action, significantly reducing pain within 48 hours. Nano-based formulations, including nano-silver and nano-CH, provided enhanced pain relief and effective microbial control while maintaining safety and reliability. Ledermix paste emerged as the most effective for rapid pain reduction in cases of acute apical periodontitis, highlighting the importance of corticosteroids and antibiotics. Advanced imaging modalities, such as cone-beam computed tomography (CBCT), facilitated accurate diagnosis and monitoring of periapical healing, demonstrating a high healing rate (76%) for large lesions treated nonsurgical.

**Conclusion:**

This review confirms the efficacy of traditional calcium hydroxide and highlights the advantages of innovative formulations such as TAPC, nanoparticles, and Ledermix paste in improving treatment outcomes. Rapid pain relief, effective microbial control, and enhanced healing underscore their potential for routine clinical use. The findings also emphasize the importance of advanced imaging and individualized treatment planning. Further research is warranted to optimize medication protocols and explore the long-term implications of emerging formulations.

## Zusammenfassung

**Hintergrund:**

Nach der Behandlung bleiben apikale Parodontitis und postoperative Schmerzen kritische Herausforderungen in der Endodontie, wobei der Erfolg oft von wirksamen intrakananikulären Medikamenten zur Kontrolle der mikrobiellen Infektion, zur Verringerung der Entzündung und zur Förderung der Gewebeheilung abhängt. Jüngste Fortschritte bei den Medikamentenformulierungen und -techniken haben die Optionen für die Behandlung dieser Erkrankungen erweitert, so dass eine systematische Überprüfung ihrer Wirksamkeit mit der Zielsetzung erforderlich ist, die Wirksamkeit verschiedener intrakananikulärer Medikamente einschließlich Calciumhydroxid (CH), dreifacher antibiotischer Paste mit Diclofenac-Kalium (TAPC), Formulierungen auf Nanobasis und Ledermix-Paste, bei der Reduzierung der mikrobiellen Belastung, der Kontrolle der Entzündung und der Linderung postoperativer Schmerzen bei endodontischen Behandlungen zu bewerten.

**Methode:**

Einschlägige Studien wurden ausgewertet, um die antimikrobielle Wirksamkeit, die Ergebnisse der Schmerzbehandlung und das Heilungspotenzial verschiedener intrakanalischer Medikamente zu bewerten. In den Studien wurden verschiedene Methoden angewandt, darunter randomisierte kontrollierte Studien und retrospektive Analysen, mit Ergebnismessungen wie Bakterienreduktion, Entzündungsmediatoren, Schmerzwerte und bildgebende Bewertung der Heilung der Läsionen.

**Ergebnisse:**

CH zeigte eine signifikante Reduktion der mikrobiellen Last (bis zu 99,5 %), verringerte Entzündungsmediatoren (IL-1β, TNF-α) und verbesserte Gewebeheilung, was es zu einer zuverlässigen Wahl für die Behandlung der apikalen Parodontitis macht. TAPC zeigte im Vergleich zu CH eine überlegene Schmerzlinderung aufgrund seiner dualen antibakteriellen und entzündungshemmenden Wirkung und reduzierte die Schmerzen innerhalb von 48 h signifikant. Formulierungen auf Nanobasis, einschließlich Nano-Silber und Nano-CH, boten eine verbesserte Schmerzlinderung und eine wirksame mikrobielle Kontrolle bei gleichzeitiger Sicherheit und Zuverlässigkeit. Ledermix-Paste erwies sich als das wirksamste Mittel zur schnellen Schmerzlinderung bei akuter apikaler Parodontitis, was die Bedeutung von Kortikosteroiden und Antibiotika unterstreicht. Moderne bildgebende Verfahren wie CBCT erleichterten die genaue Diagnose und Überwachung der periapikalen Heilung und zeigten eine hohe Heilungsrate (76 %) bei großen, nicht chirurgisch behandelten Läsionen.

**Schlussfolgerung:**

Das Review bestätigt die Wirksamkeit von herkömmlichem CH und hebt die Vorteile innovativer Formulierungen wie TAPC, Nanopartikel und Ledermix-Paste bei der Verbesserung der Behandlungsergebnisse hervor. Schnelle Schmerzlinderung, wirksame mikrobielle Kontrolle und verbesserte Heilung unterstreichen ihr Potenzial für den klinischen Routineeinsatz. Die Ergebnisse unterstreichen auch die Bedeutung einer modernen Bildgebung und einer individuellen Behandlungsplanung. Weitere Forschungsarbeiten sind erforderlich, um die Medikamentenprotokolle zu optimieren und die langfristigen Auswirkungen der neuen Formulierungen zu untersuchen.

## Introduction

### Background

Post-treatment apical periodontitis and postoperative pain are significant challenges in endodontics, often impacting patient outcomes and satisfaction. These complications arise from persistent microbial infections, unresolved inflammation, or tissue damage during or after root canal treatment. Effective management of these conditions is critical to ensuring the success of endodontic therapy, particularly in cases involving large periapical lesions, necrotic teeth, or previously failed treatments. One of the key strategies in addressing these challenges is the use of intracanal medications, which play a vital role in microbial control, inflammation management, and promotion of periapical healing.

### Microbial control and the role of intracanal medications

The primary cause of post-treatment apical periodontitis is microbial persistence or reinfection of the root canal system. Microorganisms such as Enterococcus faecalis and biofilm-forming bacteria are notoriously resistant to standard chemomechanical debridement techniques, necessitating the use of intracanal medications with strong antimicrobial properties. Calcium hydroxide (CH), a long-standing gold standard in endodontics, has been widely used for its ability to create an alkaline environment that inhibits bacterial survival. However, its slow mechanism of action and limited efficacy against certain microorganisms have prompted the exploration of alternative formulations.

### Innovations in intracanal medications

Recent advancements in intracanal medications, including triple antibiotic paste (TAP) combined with anti-inflammatory agents such as diclofenac potassium, and nano-based formulations like nano-silver (nano-Ag) and nano-calcium hydroxide (nano-CH), offer promising alternatives. However, it is critical to be aware that any local application of antibiotics should be avoided due to the risk of resistance development and is only acceptable if no antiseptic with analogous efficacy is available. These formulations aim to enhance microbial eradication while addressing inflammation, a key factor in postoperative pain and tissue healing. Triple antibiotic paste has demonstrated broad-spectrum antibacterial activity, while the addition of anti-inflammatory agents provides rapid pain relief by suppressing periradicular inflammation. Similarly, nanoparticles offer superior surface area, enhanced penetration, and improved efficacy, making them an attractive option for endodontic applications.

### Postoperative pain management and patient comfort

Postoperative pain is a common concern following root canal treatment, often resulting from mechanical, chemical, or microbial irritation of periradicular tissues. Effective pain management is critical to improving patient comfort and compliance with treatment. Traditional intracanal medications such as CH have shown limited immediate pain relief, whereas newer formulations, e.g., TAP with anti-inflammatory drugs and nano-based medications, have demonstrated superior outcomes in reducing pain within the first 48–72 hours postoperatively. Additionally, corticosteroid-based medications (e.g., Ledermix paste) have proven particularly effective in cases of acute apical periodontitis, emphasizing the role of anti-inflammatory and analgesic components in intracanal medications.

### The role of imaging in assessing outcomes

Advancements in imaging technologies, such as cone-beam computed tomography (CBCT), have revolutionized the assessment of periapical lesions and treatment outcomes. CBCT provides detailed three-dimensional evaluations of lesion size, volume, and healing progress, offering valuable insights into the efficacy of intracanal medications. Studies have demonstrated the utility of CBCT in monitoring periapical healing and guiding clinical decision-making, particularly in cases with large or complex lesions.

Despite the extensive research on intracanal medications, variations in their clinical performance and the emergence of innovative formulations necessitate a comprehensive evaluation. This review aims to compare the efficacy of traditional and novel intracanal medications, including CH, TAP, nanoparticles, and Ledermix paste, in reducing microbial loads, managing postoperative pain, and promoting periapical healing. By synthesizing the findings from recent studies, this review seeks to provide evidence-based insights into the clinical utility of these medications and identify potential areas for future research.

## Method

### Search strategy

A systematic search was conducted using electronic databases (PubMed, Scopus, Cochrane Library) for studies published between January 2020 and December 2024. Keywords used included “intracanal medication duration”, “periapical abscess”, “calcium hydroxide”, “chlorhexidine”, and “healing outcomes”. Additional manual searches were conducted in relevant journals and reference lists of selected articles. Studies were included if they:

1. Investigated the relationship between intracanal medication duration and healing outcomes of periapical abscesses
2. Reported on clinical, radiographic, or microbiological endpoints
3. Were randomized controlled trials (RCT), cohort studies, or case-control studies
4. Were published in English.

Exclusion criteria were

1. Studies with incomplete data
2. Non-comparative studies or reviews
3. Animal studies or in-vitro experiments.

### Data extraction and analysis

Data were extracted using a standardized form, including study design, sample size, intracanal medication type, duration of application, outcome measures, and results. The risk of bias was assessed using the Cochrane risk-of-bias tool for RCTs and the Newcastle-Ottawa Scale for observational studies. Meta-analysis was not performed due to heterogeneity in study designs and outcome measures.

## Results

Out of 342 records identified, 5 studies met the inclusion criteria. The key findings are summarized in Table 1 (Tab. 1).

## Discussion

The findings of the study by Barbosa-Ribeiro et al. [1] highlight the significant therapeutic potential of CH-based intracanal medication (ICM) in managing teeth with failed root canal treatments and apical periodontitis. The primary objective was to evaluate the in-vivo effects of this medication on bacterial reduction, pro-inflammatory cytokines (PICs), and matrix metalloproteinases (MMPs), which are critical markers of infection and inflammation. The results demonstrated a remarkable 99.5% reduction in culturable bacterial counts after 30 days of CH-based ICM application, reinforcing its efficacy as an antimicrobial agent. This aligns with previous studies that have reported the alkaline pH of CH as a key factor in its antimicrobial activity, as it disrupts bacterial cellular metabolism and inactivates endotoxins. In addition to bacterial reduction, significant decreases in the levels of PICs, specifically IL-1β and TNF-α, were observed post-treatment. These cytokines play a pivotal role in mediating inflammation and tissue destruction in apical periodontitis. Their reduction suggests that CH not only combats microbial infection but also modulates the host immune response, thereby creating a more conducive environment for tissue healing. The study also analyzed the impact of ICM on MMPs, which are enzymes involved in the degradation of extracellular matrix components and are often upregulated in inflammatory conditions like apical periodontitis. A decrease in MMP-2, MMP-3, MMP-8, and MMP-9 levels post-treatment reflects the potential of CH to mitigate tissue breakdown and support periradicular tissue repair. However, the observed increase in MMP-13 levels warrants further investigation. It is hypothesized that the elevation of MMP-13 could be part of a repairmechanism or an unintended inflammatory response triggered by the alkaline nature of the ICM. Interestingly, both auxiliary irrigants used during chemomechanical preparation, i.e., 2% CHX gel and 6% NaOCl, demonstrated comparable outcomes when combined with CH. This suggests that while the choice of irrigant influences initial microbial reduction, the prolonged effects of CH in intracanal medication remain consistent irrespective of the auxiliary chemical agent used. From a clinical perspective, these findings underscore the importance of incorporating CH-based ICM into treatment protocols for teeth with persistent infections and apical periodontitis. The significant reductions in microbial load, inflammatory mediators, and matrix-degrading enzymes translate to an improved prognosis for such cases. Additionally, the comparable efficacy of CHX and NaOCl provides flexibility in selecting an irrigant based on clinical preference and patient-specific considerations. The study validates the multifaceted role of CH as a vital component in endodontic treatment, extending its benefits beyond mere microbial control to include anti-inflammatory and tissue-preserving effects. However, further research is recommended to explore the mechanisms underlying the increase in MMP-13 levels and to optimize treatment protocols for maximizing therapeutic outcomes [1].

The study by Fahim et al. [2] highlights the significant potential of nano-Ag and nano-CH as intracanal medications (ICM) in endodontic retreatments, showcasing their ability to provide comparable antibacterial effects to conventional CH while offering superior post-operative pain relief. Both nano-Ag and nano-CH effectively reduced microbial counts, including *E. faecalis*, and diminished biofilm-forming capabilities, although the most pronounced microbial reduction was observed after chemomechanical debridement, reinforcing the pivotal role of cleaning and shaping as the primary intervention. The comparable antibacterial efficacy among the tested medications suggests that all are effective in inhibiting microbial activity in the root canal system. Notably, nano-Ag and nano-CH demonstrated significantly better pain control at 48 and 72 hours post-treatment compared to CH, likely due to their enhanced anti-inflammatory properties and superior penetration into dentinal tubules. The incidence of flare-ups was consistent across all groups, confirming the safety of these nanomaterials in managing post-operative complications. These findings underscore the clinical relevance of nanoparticles in enhancing patient comfort and satisfaction, particularly in cases where pain management is a priority. While the results affirm the therapeutic promise of nano-Ag and nano-CH, further research is necessary to elucidate the mechanisms driving their enhanced analgesic effects and to evaluate their long-term biocompatibility and efficacy. The study establishes a strong foundation for the integration of nanotechnology into endodontic treatments, combining effective microbial control with improved patient outcomes.

The Mosquera-Barreiro et al. study [3] provides valuable insights into the healing process of large periapical lesions treated nonsurgically, showing a high overall healing rate of 76% with a mean healing time of 19 months. Patient age and initial lesion volume were significant factors influencing healing time, with older age associated with slower bone regeneration and larger lesions requiring extended resolution due to greater bone destruction. Advanced imaging, particularly CBCT, was instrumental in accurately assessing lesion volume and monitoring healing, emphasizing its clinical utility. Interestingly, gender, treatment type, and filling material did not significantly affect outcomes, underscoring the importance of the clinician’s skill and adherence to protocols. Clinicians should set realistic expectations for older patients and those with extensive lesions and consider periodic CBCT monitoring for precise evaluation. While the study’s retrospective design and lack of systemic health data are limitations, it highlights key predictors of healing time and supports the integration of CBCT to enhance diagnostic accuracy and patient care.

Ehrmann et al. [4] investigated the relationship between postoperative pain and three different intracanal medications placed after biomechanical debridement in patients presenting with pulp necrosis and acute apical periodontitis. A total of 223 teeth from 221 patients seeking emergency pain relief at the Royal Dental Hospital of Melbourne were included. All teeth underwent conventional root canal treatment using the step-back technique with hand files, irrigated with 1% NaOCl followed by 15% ethylenediaminetetraacetic acid (EDTAC). The canals were dried and randomly assigned to one of three groups: Group 1 received Ledermix paste, Group 2 was treated with CH paste, and Group 3 had no dressing. Patients reported preoperative pain levels using a visual analog scale, and recorded postoperative pain levels 4 hours after treatment as well as daily for 4 days. The results revealed that the mean preoperative pain score for all groups was between 42 and 48 hours. After 4 days, pain scores were significantly lower in Group 1 (mean score of 4), compared to Group 2 (mean score of 10) and Group 3 (mean score of 7). Group 1 exhibited a statistically significant reduction in postoperative pain (P=0.04), while no significant difference was observed between Groups 2 and 3. Overall, the mean preoperative pain score of 44.4 for all groups dropped by 50% to 22.1 within 24 hours. The rapid and sustained pain reduction observed with Ledermix underscores its effectiveness as an intracanal medication in managing postoperative pain associated with acute apical periodontitis. The superior pain relief achieved with Ledermix can be attributed to its corticosteroid and antibiotic components, which likely reduce inflammation and suppress microbial activity more effectively than CH or no dressing. CH, while effective in creating an alkaline environment unfavorable to bacteria, did not achieve the same level of immediate pain relief, likely due to its slower mechanism of action. Interestingly, the absence of a dressing did not significantly alter pain outcomes compared to CH, highlighting the critical role of complete biomechanical debridement in pain reduction. These findings have important clinical implications. They emphasize the value of Ledermix as an intracanal medication for rapid postoperative pain control in patients with acute apical periodontitis, particularly in emergency settings. Furthermore, the study reinforces the importance of thorough biomechanical preparation as a cornerstone of endodontic pain management. Future research should explore additional variables, such as the impact of systemic health conditions or variations in medication formulations, to further optimize postoperative pain control strategies.

The study by Omaia et al. [5] investigated the effectiveness of CH and triple antibiotic paste with diclofenac potassium (TAPC) in reducing postoperative pain in patients with asymptomatic uniradicular necrotic teeth. Flare-ups following root canal treatment, characterized by the acute exacerbation of asymptomatic pulpal or periradicular conditions, are often attributed to mechanical, chemical, or microbial irritation. Given the critical role of microorganisms in interappointment pain, successful endodontic treatment hinges on their complete eradication through mechanical cleaning, shaping, irrigation, and the use of effective antibacterial agents. The study involved 84 patients randomly assigned to either the CH or TAPC group. After intracoronal cavity preparation and chemomechanical debridement with rotary Protaper Universal files and saline irrigation, intracanal medications were placed, and postoperative pain was assessed using a VAS at 24, 48, and 72 hours. Both medications significantly reduced postoperative pain over time, demonstrating their efficacy in managing pain associated with asymptomatic uniradicular necrotic teeth. However, TAPC showed a superior ability to reduce pain compared to CH, with a statistically significant difference observed at 48 hours. The enhanced performance of TAPC may be attributed to its combination of antibacterial properties and anti-inflammatory effects from diclofenac potassium, which likely suppresses microbial activity while mitigating periradicular inflammation. Calcium hydroxide, a widely used intracanal medication known for its antimicrobial properties and ability to create an alkaline environment unfavorable to bacterial survival, was effective but lacked the added anti-inflammatory benefits of TAPC. These findings have practical implications for clinical practice. Both medications are effective in reducing postoperative pain; however, TAPC may be the preferred choice in cases where rapid pain relief is a priority, particularly in patients prone to flare-ups. The results also emphasize the importance of incorporating anti-inflammatory agents in intracanal medications to enhance patient comfort and treatment outcomes. Future research should explore the long-term clinical outcomes and potential effects of TAPC on tissue healing and regeneration, as well as the role of other anti-inflammatory agents in reducing postoperative endodontic pain.

## Conclusion

The review highlights the significant advancements in intracanal medications and techniques. CH remains a reliable intracanal medication, demonstrating its antimicrobial efficacy with substantial reductions in bacterial counts, pro-inflammatory cytokines, and matrix metalloproteinases, leading to enhanced tissue healing. However, innovative approaches, such as the use of triple antibiotic paste with diclofenac potassium (TAPC) and nano-based medications, have shown superior outcomes in pain management due to their combined antibacterial and anti-inflammatory properties. TAPC and nanoparticles such as nano-silver and nano-CH offer enhanced patient comfort by providing rapid and effective pain relief while maintaining microbial control. Additionally, Ledermix paste has proven to be particularly effective for rapid pain reduction in acute apical periodontitis due to its corticosteroid and antibiotic components.

The review underscores the importance of thorough biomechanical debridement and the use of advanced imaging modalities, such as CBCT, for accurate diagnosis and monitoring of lesion resolution, especially in cases with large periapical lesions. Clinical practice should prioritize medication selection based on individual patient needs, focusing on rapid pain relief, microbial eradication, and long-term healing potential. Furthermore, the findings emphasize the need for continued research into the role of advanced formulations, including the long-term effects of nanoparticles and the implications of increased MMP-13 levels observed in certain treatments. Ultimately, the integration of these innovations into routine clinical practice holds promise for improving treatment outcomes and patient care in endodontics.

## Notes

### Authors’ ORCIDs

- Karthik Shunmugavelu: 0000-0001-7562-8802
- Vimala Rani Swaminathan: 0000-0002-4938-0943

### Funding

None. 

### Competing interests

The authors declare that they have no competing interests.

## Figures and Tables

**Table 1:**
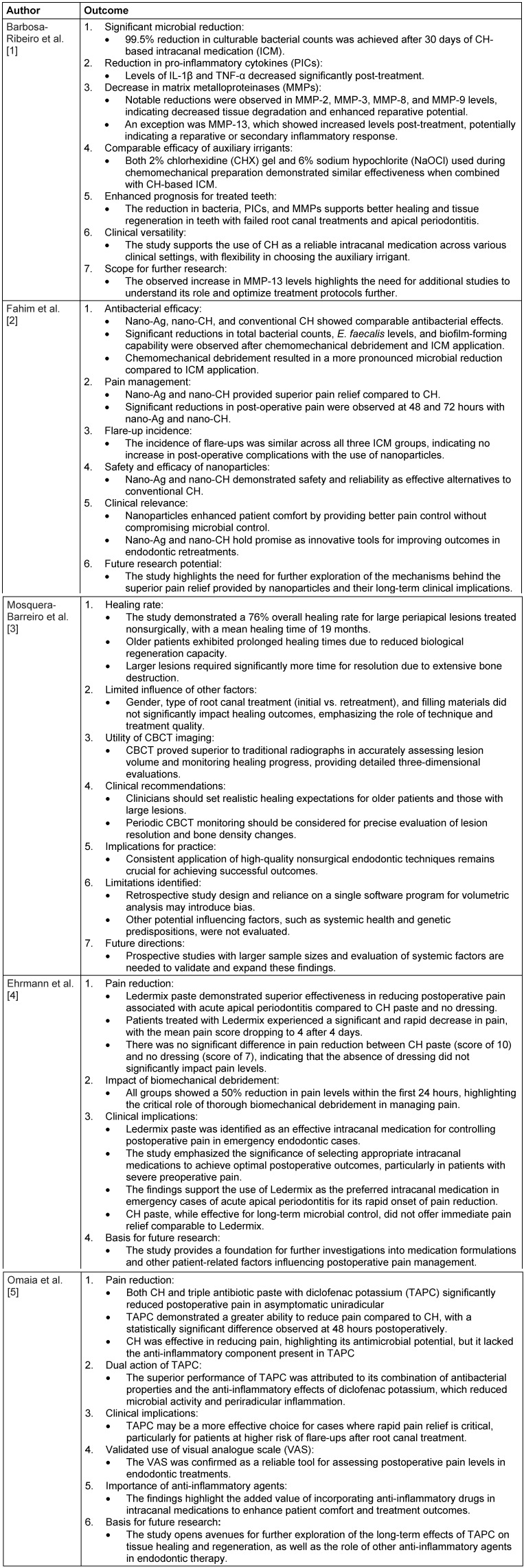
Key findings of the five studies analyzed
